# Tibialis Posterior Tenosynovitis and Associated Pes Plano Valgus in Rheumatoid Arthritis: Electromyography, Multisegment Foot Kinematics, and Ultrasound Features

**DOI:** 10.1002/acr.21859

**Published:** 2013-03-28

**Authors:** Ruth Barn, Deborah E Turner, Daniel Rafferty, Roger D Sturrock, James Woodburn

**Affiliations:** 1 School of Health and Life Sciences, Glasgow Caledonian UniversityGlasgow, UK; 2 Glasgow Royal InfirmaryGlasgow, UK

## Abstract

**Objective:**

To compare electromyographic (EMG), kinematic, kinetic, and ultrasound (US) features of pes plano valgus associated with US-confirmed tibialis posterior (TP) tenosynovitis in rheumatoid arthritis (RA) and healthy control subjects.

**Methods:**

In this cross-sectional study, patients with RA and US-confirmed tenosynovitis of TP underwent gait analysis, including 3-dimensional kinematics, kinetics, and intramuscular EMG of TP, and findings were compared with a group of healthy individuals. The RA group also underwent B mode and power Doppler US scanning of the TP tendon to assess and score levels of pathology.

**Results:**

Ten patients with RA, median (range) disease duration of 3 years (1–18 years), and 5 control subjects were recruited. Compared to control subjects, the RA patients walked slower and presented with moderate levels of foot-related disability. The mean ± SD Disease Activity Score in 28 joints was 4.6 ± 1.6. Increased magnitude of TP activity was recorded in the RA group compared to controls in the contact period of stance (*P* = 0.007), in conjunction with reduced ankle joint power (*P* = 0.005), reduced navicular height in the medial arch (*P* = 0.023), and increased forefoot dorsiflexion (*P* = 0.027). TP tendon thickening, fluid, and power Doppler signal were observed in the majority of patients.

**Conclusion:**

This study has demonstrated, for the first time, increased TP EMG activity in the presence of US-confirmed TP tenosynovitis in RA. Altered muscle function occurred in conjunction with suboptimal mechanics, moderate levels of tendon pathology, and active disease. Targeted therapy may be warranted to reduce inflammation and mechanically off-load diseased tendon states.

## INTRODUCTION

Rheumatoid arthritis (RA) is an inflammatory polyarthritis that frequently affects the joints and soft tissues of the feet (1). Tibialis posterior (TP) tenosynovitis has a reported prevalence between 13–64% in RA, dependent upon the diagnostic criteria employed (2). The condition is associated with a progressive flat foot deformity (pes plano valgus [PPV]) and significant walking-related disability (2). Both mechanical and inflammatory factors have been postulated in the development of this complex clinical problem (3), but definitive data are lacking. Furthermore, the functional contribution of the TP muscle when PPV and TP tenosynovitis coexist is not known.

TP activity in RA has previously been investigated using intramuscular electromyography (EMG) (4). The results demonstrated increased TP activity in an RA group with PPV compared to those without (4). However, the study was conducted in patients with longstanding disease duration and the pathologic state of the tendon was unknown. Similar results have been reported in flat foot (5) and TP tendon dysfunction patients without RA (6), with both studies concluding that the increased activity occurred in an attempt to prevent collapse of the medial longitudinal arch (MLA). In addition to alterations to muscle activity, PPV in RA is associated with structural and functional deterioration of the rear- and midfoot joints (7–9). In an RA population, TP tendon disease and PPV frequently coexist, yet the relationship between the two remains ambiguous. Some authors speculate that soft tissue changes to the TP tendon and laxity of supporting structures cause the valgus rearfoot alignment (10–12). Others suggest that subtalar and midfoot arthritis and synovitis in the context of weight-bearing stresses are more likely to be the cause (11, 13–16). Recent advances in imaging and multisegment foot models represent an opportunity to better understand the condition by combining biomechanical features with imaging of tendon pathology in RA.

**Significance & Innovations**

- Innovative use of ultrasound (US) technology permits fine-wire electromyographic (EMG) studies of tibialis posterior function during gait.
- This study provides a novel, detailed description of mechanical and inflammatory factors in US-confirmed tibialis posterior tenosynovitis in rheumatoid arthritis (RA).
- This is the first study to demonstrate abnormal tibialis posterior EMG activity in a cohort of patients with RA and US-confirmed tibialis posterior tenosynovitis; this was observed in the presence of suboptimal biomechanics and moderate levels of tendon disease.

High-resolution ultrasound (US) has been reported as the gold standard for the investigation of tendons (17–19). US facilitates detailed examination of tendon features, including assessment of internal structure of tendon body, tendon sheath, and the presence of hyperemia suggestive of active inflammation via color or power Doppler signal (PDS) (20, 21). The aim of this study is to provide a comprehensive description of the biomechanical and inflammatory features of TP tenosynovitis in RA by combining EMG with 3-dimensional (3-D) motion analysis and high-resolution US. These features were compared to healthy individuals for analysis.

## PATIENTS AND METHODS

### Patients

Patients were recruited from outpatient clinics at Glasgow Royal Infirmary and Gartnavel General Hospital, Glasgow, UK. Patients were eligible for inclusion if they had a confirmed diagnosis of RA based on the 1987 American College of Rheumatology criteria (22), passively correctable PPV deformity, and US-confirmed tenosynovitis at a screening appointment. PPV is a complex multiplanar deformity with the following features: valgus rearfoot alignment, MLA collapse, and medial bulging of the talonavicular joint (4, 23), in conjunction with abduction of the forefoot (8). Patients exhibiting these features in relaxed standing were included in the study. Tenosynovitis was defined as “hypoechoic or anechoic thickened tissue with or without fluid within the tendon sheath which may or may not exhibit Doppler signal” (24). Presence was confirmed by diagnostic US prior to entry to the study. Control subjects were recruited from Glasgow Caledonian University staff. Subjects were included if they had no history of previous or current musculoskeletal or neurologic disease affecting the lower leg and absence of foot pain and deformity. The study was conducted in accordance with the Declaration of Helsinki, and ethical approval was obtained from the West of Scotland Local Research Ethics Committee and NHS Greater Glasgow and Clyde Research and Development.

### Demographic, disease, and clinical assessment

The participants' age, sex, and disease duration were recorded. A core set of clinical variables were recorded: tender and swollen foot joint count undertaken by a single clinician (RB); foot posture using the Structural Index (16); foot-related impairment and disability using the Foot Impact Scale for RA (25); and global disability using the Health Assessment Questionnaire (26). Disease activity was recorded using a composite measure, the Disease Activity Score in 28 joints (DAS28) (27), including erythrocyte sedimentation rate within 2 weeks of assessment. Visual analog scales (100 mm) were used to record foot pain, general health, and arthritis pain. The most symptomatic leg was studied in the RA group; in the control group the studied leg was randomly selected by the participant selecting a number between 1 and 10, then was randomly assigned to the right or left leg.

### Biomechanical analysis

A 12-camera 120 Hz, 3-D motion analysis system (Qualisys Oqus) was used to track the motion during the gait of a multisegmented foot model comprising functional units for the shank, rearfoot, midfoot, and forefoot (28). A single force plate (Kistler) recorded ground reaction forces simultaneously. Visual 3-D software (C-Motion) was used to extract a core set of functional variables: peak ankle joint moments and power, peak rearfoot eversion, midfoot inversion, forefoot abduction, forefoot dorsiflexion, and lowest navicular height. Walking speed was self-selected and recorded using timing gates (Brower Timing Systems). Trials exceeding ±5% of the self-selected speed were excluded, and a total of 5 walking trials were included for each participant.

### EMG analysis

Four channels of surface EMG data were recorded for tibialis anterior, soleus, peroneus longus, and medial gastrocnemius using Trigno (Delsys) wireless surface electrodes applied following the Surface ElectroMyoGraphy for the Non-Invasive Assessment of Muscles guidelines (29). Surface electrodes had a single differential configuration, interelectrode distance of 10 mm, 4-bar formation, bandwidth of 20–450 Hz, and 99.9% silver contact material. Intramuscular EMG of TP was undertaken using bi-polar stainless-steel nylon-coated fine wire electrodes (Motion Lab Sytems). Electrodes were inserted under US guidance (Esaote Mylab 70) using a 13–4-MHz linear array transducer via the posterior-medial approach at 50% of the distance between the medial malleolus and the tibial tubercle (30). Placement of the electrode was verified by checking the signal while applying manual resistance in the direction of dorsiflexion and eversion while participants plantarflexed and inverted; the signal was also checked when participants flexed their toes to ensure the electrode was not placed in the flexor digitorum longus muscle. Discrete variables were recorded for each muscle relating to the peak of activity and the time of peak activity during contact and combined midstance/propulsive (MS/P) phases of stance, based on when the muscles were most active (31).

### US assessment of tenosynovitis

High-resolution US was undertaken by a single experienced sonographer (DET) using an Esaote MyLab 70 with 15–7-MHz linear array transducers. TP was viewed and images recorded along the length of the tendon at 3 locations: medial malleolus, navicular insertion, and midway between the 2 points. Measurements of tendon diameter and fluid were recorded in the retro malleolar region and compared with published literature (32, 33). PDS was recorded using a pulse repetition frequency of 750 Hz and the Doppler gain was optimized to regional site (34). The levels of PDS were graded using a 4-point semiquantitative scale (absent/minor/moderate/major) (35). Only the RA group underwent US scanning of TP; normative values for tendon diameter and fluid based on the work of Schmidt et al (33) and Premkumar et al (32) were used for comparison.

### Data processing

All EMG signals were high-pass filtered with a cutoff frequency of 20 Hz. All EMG data were subject to a root mean squared moving average of 25 msec. EMG data were normalized to maximum voluntary isometric contractions (MVICs); 3 MVICs were recorded for each muscle following completion of walking trials. The MVIC data were recorded for 5 seconds with a gradual buildup of 2 seconds prior to maximal effort for the final 3 seconds. The peak value from a 500 msec window obtained from the 3-second maximal effort of the MVIC was used as the reference value, similar to the methods reported elsewhere (36). All participants were verbally encouraged in a standard manner during the MVICs, and a 1-minute recovery period was set between repetitions. Kinematic data were subject to a fourth-order Butterworth low-pass filter with a cutoff of 6 Hz.

### Statistical analysis

Statistical analyses were performed using SPSS, version 17.0. Demographic and group characteristics were summarized with the mean and SD or median and range. Biomechanical and EMG data were normalized to 100% of stance and compared using the Student's *t*-test or Mann-Whitney U test, according to the distribution characteristics of the data.

## RESULTS

### Group characteristics

Ten patients (6 women, 4 men), with a mean ± SD age of 50 ± 9 years and a median (range) disease duration of 3 years (1–18 years), were recruited (Table 1). Five control subjects, with a mean ± SD age of 47 ± 6 years, were also recruited. Patients with RA and TP tenosynovitis walked on average 20% slower than the control group and had moderate levels of foot-related impairment and disability. Demographics of the groups were comparable with the exception of body mass index (BMI); in the RA group, 2 participants were within the ideal range, 3 were overweight, and 5 were obese. In the control group, 3 participants were within the ideal range and 2 were overweight. All patients with RA were managed on disease-modifying antirheumatic drug therapy and 2 patients were receiving biologic drug therapy. Moderately active disease states were present in the RA cohort with a mean ± SD DAS28 score of 4.6 ± 1.6.

**Table 1:** Demographic and disease characteristics*

Variable	RA group (n = 10)	Control group (n = 5)
Age, years	50 ± 9	47 ± 6
Sex, M:F	4:6	2:3
Disease duration, median (range) years	3 (1–18)	–
Body mass index, kg/m^2^	30 ± 6	24 ± 1
DAS28 score	4.6 ± 1.6	–
FIS impairment subscale (range 0–21)	14 ± 3	0 ± 1
FIS disability subscale (range 0–30)	21 ± 5	0 ± 0
HAQ score	1.3 ± 0.6	0 ± 0
Foot pain VAS (0–100 mm)	46 ± 19	1 ± 1
General health VAS (0–100 mm)	44 ± 26	1 ± 2
Arthritis VAS (0–100 mm)	51 ± 19	–
Structural index: rearfoot (range 0–7)	2 ± 1	1 ± 1
Structural index: forefoot (range 0–12)	4 ± 3	3 ± 3
Swollen foot joint count (range 0–14)	0 ± 1	0 ± 0
Tender foot joint count (range 0–14)	7 ± 3	0 ± 0
Barefoot walking speed (meters/second)	1.00 ± 0.14	1.25 ± 0.15
Weight-bearing rearfoot alignment, degrees†	−7 ± 3	−4 ± 2

* Values are the mean ± SD unless indicated otherwise. RA = rheumatoid arthritis; DAS28 = Disease Activity Score in 28 joints; FIS = Foot Impact Scale for RA; HAQ = Health Assessment Questionnaire; VAS = visual analog scale.

† By convention, eversion angles are expressed as negative.

### Biomechanical features

**Figure 1:**
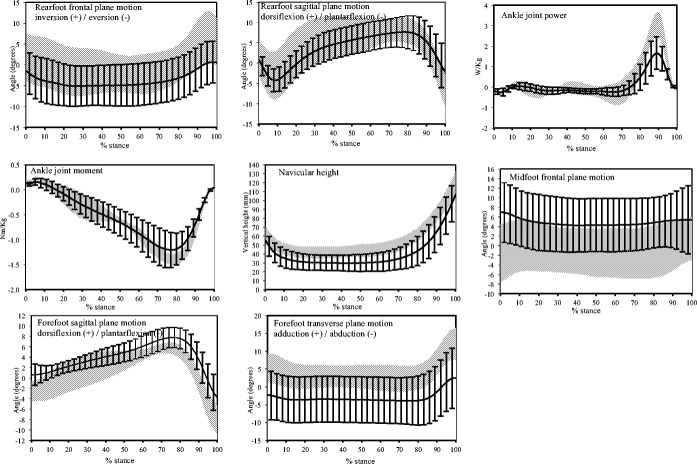
Motion and force time curves. Shaded area shows the mean ± SD for 5 control participants; bars show the mean ± SD for 10 rheumatoid arthritis patients.

In comparison to healthy control subjects, the RA group demonstrated a trend towards abnormal intersegment foot motion and force in the presence of slower walking speed. The RA group demonstrated a trend towards characteristic features of PPV: reduced medial longitudinal arch height (planus), increased rearfoot eversion (valgus), and forefoot abduction (Table 2 and Figure 1). However, when key discrete variables were compared between the groups, only 3 of 8 variables had a *P* value less than 0.05 and the 95% confidence interval of the mean difference for the remaining variables crossed zero. The 3 variables were reduced ankle joint power, lower navicular height, and increased peak forefoot dorsiflexion compared to controls.

**Table 2:** Key kinematic and kinetic variables*

Segment and variable	RA barefoot (n = 10)	Control barefoot (n = 5)	Mean difference (95% CI)	*P*†
Rearfoot				
Peak eversion, degrees	−5 ± 5	−3 ± 5	−2 (−8, 4)	0.53
Peak plantarflexion, degrees	−6 ± 3	−7 ± 4	2 (−3, 6)	0.44
Peak ankle joint power, W/kg	1.7 ± 0.8	3.1 ± 0.6	−1 (−2, 0)	0.005‡
Peak ankle joint moment, Nm/kg	−1.2 ± 0.3	−1.4 ± 0.1	0.2 (0, 0.5)	0.11
Midfoot				
Lowest navicular height, mm	29 ± 9	41 ± 7	−12 (−22, −2)	0.02‡
Peak inversion, degrees	7 ± 6	2 ± 5	6 (−1, 13)	0.08
Forefoot				
Peak abduction, degrees	−5 ± 7	2 ± 4	−6 (−13, 1)	0.07
Peak dorsiflexion, degrees	8 ± 2	6 ± 1	3 (0, 5)	0.02‡

* Values are the mean ± SD unless otherwise indicated. RA = rheumatoid arthritis; 95% CI = 95% confidence interval.

† By independent-samples *t*-test.

‡ Significant at *P* < 0.05.

### EMG features

**Figure 2:**
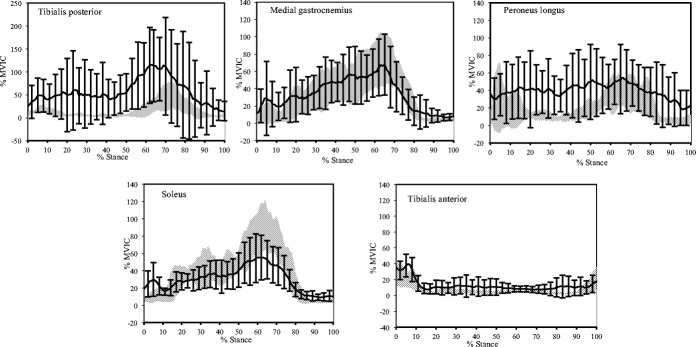
Electromyography activation profiles. Data expressed relative to maximum voluntary isometric contractions (MVICs) during the stance phase. Shaded area shows the mean ± SD for 5 control participants; bars show the mean ± SD for 10 rheumatoid arthritis patients.

There was a trend for increased EMG activity of the TP and tibialis anterior muscles and reduced soleus activity in the RA group compared to controls. EMG data were not normally distributed (9 variables negatively skewed, 5 variables positively skewed) and are summarized accordingly in Table 3 and Figure 2. There was also evidence of altered TP timing, which is suggestive of earlier peak of activity in the contact phase and later peak of activity in the MS/P phase, and a trend towards earlier peak of soleus activity but with reduced magnitude. Magnitude of TP in the contact phase, timing of TP during contact and MS/P, and timing of soleus during MS/P had significance values of *P* < 0.05. However, when adjusted for multiple testing these were no longer significant.

**Table 3:** Key discrete EMG variables*

Muscle and variable	RA barefoot (n = 10)	Control barefoot (n = 5)	*P*†
Medial gastrocnemius			
Peak MS/P	83 (59–128)	81 (65–106)	1.00
Time of peak MS/P	46 (34–65)	63 (52–69)	0.19
Peroneus longus			
Peak contact	43 (28–86)	19 (6–65)	0.12
Time of peak contact	9 (5–15)	5 (4–12)	0.35
Peak MS/P	70 (43–105)	39 (36–59)	0.11
Time of peak MS/P	68 (38–77)	69 (56–78)	0.75
Soleus			
Peak MS/P	69 (31–84)	95 (68–123)	0.08
Time of peak MS/P	61 (48–63)	64 (63–67)	0.04‡
Tibialis anterior			
Peak contact	49 (32–56)	27 (16–44)	0.07
Time of peak contact	6 (0–6)	0 (0–8)	0.94
Tibialis posterior			
Peak contact	48 (35–116)	22 (14–28)	0.007‡
Time of peak contact	13 (8–15)	7 (5–8)	0.03‡
Peak MS/P	94 (56–261)	51 (22–80)	0.06
Time of peak MS/P	64 (60–68)	74 (72–75)	0.01‡

* Values are the median (interquartile range) unless indicated otherwise. Magnitude data expressed as percentage of maximum voluntary isometric contractions; temporal data expressed as percentage stance. EMG = electromyography; RA = rheumatoid arthritis; MS/P = combined midstance/propulsive phase gait.

† By Mann-Whitney U test.

‡ Significant at *P* < 0.05.

### US features

Measurement of TP tendon diameter was recorded in the transverse and longitudinal views at the medial malleolus level, and the longitudinal:transverse ratio was calculated. Additionally, fluid was measured in both views; all data were normally distributed and values are summarized in Table 4 as mean ± SDs. The range of values is also included to further describe the cohort. Compared to normal values from the literature, the TP tendon was thickened in the longitudinal view, and levels of fluid were elevated in the patients with RA and TP tenosynovitis. Levels of PDS were also recorded at 3 sites; all participants had confirmed PDS in 1 or more sites. The greatest level of pathology was recorded at the navicular insertion region, where 5 of 10 scored as moderate, 1 of 10 as major, 1 of 10 as minor, and 3 of 10 as absent.

**Table 4:** Ultrasound features of the tibialis posterior (TP) tendon*

	RA cohort (n = 10)		Published normal values	
		
Variable	Mean ± SD	Range	Mean ± SD†	Range
TP transverse, mm	9.4 ± 0.9	7.4–10.9	8.4 ± 4.2	3.1–14.1
TP longitudinal, mm	4.9 ± 1.1	3.0–6.0	2.8 ± 1.8	1.3–6.0
Ratio longitudinal:transverse	0.53 ± 0.12	0.30–0.64	0.30 ± 0.14	0.20–0.46
Fluid transverse, mm	2.3 ± 1.6	0.7–4.9	1.2 ± 1.6	0.2–3.8
Fluid longitudinal, mm	1.3 ± 1.0	0.0–2.8		

* Published values from 102 control subjects (33); normative ratio values from 15 control subjects (32). RA = rheumatoid arthritis.

† 2 SDs.

## DISCUSSION

The aim of this study was to provide a comprehensive description of TP tenosynovitis associated with PPV in RA, including imaging of tendon pathology, and to compare these features to normal values. The current study is the first to investigate EMG activity of TP in RA-associated PPV with TP tenosynovitis confirmed by US imaging. The increased TP activity occurred in conjunction with abnormal mechanical function, moderate levels of TP tendon pathology on US, and reduced walking speed. Abnormal gait patterns and reduced walking speed have been previously reported in RA (7, 8, 16). The results of this study build upon previous findings to attempt to understand the relationship between muscle activity and joint motion and forces. The results must be considered within the context of moderate levels of foot-related impairment and disability and active disease states.

TP acts as the primary dynamic stabilizer of the rearfoot and the MLA (37, 38). Increased TP activity has been postulated as a potential mechanism to prevent collapse of the MLA in RA and non-RA flatfoot cohorts (4, 5) and a TP tendon dysfunction cohort (6). In the present study, the increased magnitude of activity was pronounced in the contact period of stance when the foot adapts to the weight-bearing surface. However, the increased activity was not sufficient to prevent midfoot collapse as demonstrated by lower navicular height. There was also evidence of altered timing of TP, which is suggestive of earlier peak of activity in the contact phase and later peak of activity in the MS/P phase, as well as a trend toward earlier peak soleus activity but with reduced magnitude. There was also a trend toward increased tibialis anterior activity in agreement with findings in flatfoot and TP tendon dysfunction cohorts (5, 6), although this did not reach statistical significance. The trend for increased tibialis anterior activity may be an attempt to assist the TP muscle to control initial rapid pronation during contact. No other abnormal muscle activation patterns were evident in this cohort. Previous studies have reported high levels of variation in EMG profiles in healthy adults (31), which may be confounded by compensatory mechanisms in RA. Intersubject variability highlights the person-specific muscle activation profiles, which in RA can be further confounded by adaptive strategies to localized joint/soft tissue pain that was reported in the cohort. Moreover, loss of muscle mass and strength associated with RA (39) may further contribute to variation.

In RA, the joints of the rear- and midfoot are vulnerable to inflammatory damage, leading to altered joint congruence, ligament and capsule damage, and instability (40). This manifests clinically as localized pain, tenderness, and deformity, and functionally as gait adaptations to off-load painful structures (41). Differences were detected in the midfoot and forefoot in this cohort compared to control subjects, in line with previous research (42), yet only mild to moderate rearfoot valgus was recorded compared to heterogeneous (3), severely deformed (8), and early RA cohorts (43). PPV is a multiplanar deformity affecting multiple segments within the foot to varying degrees. However, repeated forces applied during gait may lead to progressive deformity if left untreated (7). In the present study, reduced ankle joint power was evident in the RA group, and this can be attributed to reduced walking speed. Altered joint motion and forces may increase stress on the TP tendon, and the BMI status of the RA group may compound this factor. Furthermore, abnormal kinematics found in flatfoot has been reported to increase the length of the TP muscle (44). In conjunction with joint instability and pain in RA, these features may potentially combine to result in the complex adaptations as observed.

Stress on a tendon is related to muscle activity and tendon size (45). Therefore, increased TP activity may potentially contribute to the development of tendon disease in this population. The navicular insertion of TP has been described as an “enthesis organ” and is a known site for stress dissipation (46). Abnormal tendon loading occurs where the load is altered in terms of magnitude, frequency, direction, or duration (47). In this cohort, the greatest level of PDS was recorded in the region of the navicular insertion; conceivably, this may be linked to the increased TP activity in combination with the midfoot collapse. The retromalleolar region of the TP tendon is a known site for compressive stress, where the tendon changes direction (48, 49), and has a known component of fibrocartilage at the insertion and in the retromalleolar region (50). There was evidence of abnormal thickening and increased levels of fluid in this region compared to normal values, but the majority of subjects had either absent or minor levels of PDS. However, the role of inflammatory factors cannot be underestimated due to the moderate levels of disease activity present in the studied cohort. Synovial tissue is a primary target in RA, including the synovial lining of tendons, and the effect of globally active disease is a potentially confounding factor.

This study was subject to 4 main limitations. First, RA is a systemic disease involving synovial tissue including joints and tendons. Disease activity varied across the RA patients, and TP involvement in those with moderate to high levels of disease activity may be driven systemically with little or no mechanical involvement. The global effects of the disease are likely to contaminate the findings of detailed analysis of the foot and lower leg. Second, EMG normalization techniques present limitations in groups such as RA patients, where disease factors such as joint or tendon pain influence capability to generate MVICs. While the results are encouraging in terms of detecting a difference between the RA group and healthy controls, it is impossible to separate the contribution of the normalization method to the differences recorded. Despite the potential influence of the normalization method, no differences were recorded for the other studied muscles. Third, the small sample size does not provide adequate statistical power for robust conclusions to be drawn. This must be balanced against a complex protocol that has permitted initial and important insights into mechanical and inflammatory factors in RA. Finally, the role of other factors, particularly obesity, may confound the results and this should be considered in future studies.

In summary, this study has demonstrated increased magnitude of TP EMG in a cohort of patients with RA, PPV, and US-confirmed tenosynovitis. Both inflammatory and mechanical factors are thought to be important drivers of foot-related impairment and disability. However, previous studies have only considered one aspect, i.e., either the mechanical deficits or the frequency and distribution of inflammatory lesions. Despite a small sample size, this study shows for the first time that inflammation and mechanical dysfunction coexist, exploiting capabilities with 3-D gait analysis and US imaging. It does not infer cause and effect nor seek to make correlations between these factors. It does, however, provide important insights as the basis to encourage larger-scale studies that may influence the future development of targeted intervention.
